# Outcomes Following Hydraulic Pressure Indirect Sinus Lift in Cases of Simultaneous Implant Placement With Platelet-Rich Fibrin

**DOI:** 10.7759/cureus.28087

**Published:** 2022-08-16

**Authors:** Shivendra Choudhary, Yashika Bali, Amrit Kumar, Vaibhav Singh, Ravpreet Singh, Kamal Nayan

**Affiliations:** 1 Department of Dentistry, Patna Medical College, Patna, IND; 2 Department of Prosthodontics and Crown and Bridge, Swami Vivekanand Subharti University, Subharti Dental College & Hospital, Meerut, IND; 3 Department of Periodontology and Oral Implantology, Buddha Institute of Dental Sciences and Hospital, Patna, IND; 4 Department of Dentistry, Rajkiya Medical College, Orai, IND; 5 Department of Prosthodontics and Crown and Bridge, Baba Jaswant Singh Dental College Hospital and Research Institute, Ludhiana, IND; 6 Department of Prosthodontics and Crown and Bridge, Mithila Minority Dental College and Hospital, Darbhanga, IND

**Keywords:** simultaneous implant placement, prf, indirect sinus lift, hydraulic pressure technique, dental implants

## Abstract

Background

To achieve a better long-term prognosis in the posterior maxilla with poor quality of bone, the sinus lift must ensure bone regeneration till the apex of the dental implant for osseointegration. An indirect sinus lift is a minimally invasive procedure where simultaneous bone condensation is achieved. During the sinus lift procedures, different graft materials are used to gain the height of the bone in the sinus. The present study aimed to evaluate the outcomes of indirect sinus lift with hydraulic pressure and the simultaneous placement of implant using platelet-rich fibrin (PRF).

Methodology

In total, 24 subjects aged 18-74 years with missing maxillary premolars and first and second molars who opted for dental implants placed with indirect sinus lift with hydraulic pressure and had low sinus with less residual ridge height, bone density, and bone height were assessed at one day, one week, one month, three months, and six months.

Results

The average mean height preoperatively was 5.573 ± 0.66 mm which showed a significant increase postoperatively to 9.603 ± 0.78 mm (p < 0.001). Mean sinus membrane lift was 4.8 ± 2.2 mm at six months. The implant stability quotient increased significantly at six months postoperatively from 69.07 ± 3.39 at the immediate postoperative time to 72.92 ± 2.714 at six months postoperatively (p < 0.001).

Conclusions

The current study suggests that minimally invasive indirect sinus lift with bone augmentation utilizing PRF increased residual alveolar ridge height and implant stability with fewer problems than previous sinus lift procedures in the posterior maxillary area.

## Introduction

Recently, dental implants have become the most promising and accepted replacement for missing teeth to restore both function and form optimally. Dental implants have been used in all regions of both arches. However, the posterior maxilla offers some limitations in the placement of dental implants, including a close approximation of the alveolar bone and sinus, increased maxillary sinus pneumatization, inadequate posterior alveolus, the deficient height of the alveolar bone, and a flat palatal vault. In cases where small bone volume is to be regenerated, the maxillary sinus with intact periosteum, and Schneiderian membrane, a stable blood clot is ideal [1].

To obtain sufficient volume of bone in the atrophic posterior maxilla, the most commonly performed pre-prosthetic procedure is sinus augmentation. Lateral window osteotomy for a direct sinus lift is the most commonly used technique in the severely atrophied posterior maxilla. Despite being predictable, lateral window osteotomy has various limitations, including procedural morbidity, sinus complications, membrane perforation risk, and being invasive. An indirect sinus lift is a minimally invasive procedure where simultaneous bone condensation is achieved [2]. Newer instruments and equipment have been used for crestal sinus lift in a reproducible, successful, simple, and safe method using stoppers, special drills, hydraulic techniques, balloon elevation of the antral floor, and piezo instruments.

As it is usually atrophic, as reported in the literature, sinus floor elevation is usually done in more than half of the dental implants placed in the posterior maxilla. This atrophic maxilla and the need for the augmentation of the sinus floor can be attributed to progressive pneumatization of the maxillary sinus along with the continuous resorption of alveolar ridge in the maxilla following tooth extraction, which is seen in the apical direction. Moreover, the bone quality seen in the maxilla is usually poor compared to other parts of the oral cavity [3].

One of the most common complications encountered while performing the sinus floor elevation procedures is the perforation of the sinus membrane. The percentage of sinus membrane perforation is different with the different methods used as reported by the previous literature data. During the elevation of the sinus mucosa or while fracturing the floor of the maxillary sinus, perforations of the sinus membranes are usually performed [4].

Owing to the close proximation of the crestal bone to the sinus floor and pneumatization of the maxillary sinus in the vertical direction of the posterior edentulous maxilla, the basic need for sinus floor augmentation may be compromised. To overcome this limitation, in the mid-1970s, sinus floor elevation or sinus lift procedures were introduced in the field of dentistry. The aim of these sinus elevation techniques was to elevate the maxillary sinus membrane to create a sub-antral space to increase the height of the alveolar bone vertically [5].

Different types of bone grafts are being used for the reconstruction of the posterior atrophic maxilla. These bone grafts include synthetic biomaterials, heterogeneous bone grafts, homogeneous bone grafts, and autogenous bone grafts. Better knowledge and understanding, along with an accurate diagnosis concerning bone remodeling in the posterior maxillary arch, can have high value in the precise placement of dental implants. Hence, the success of the sinus augmentation and sinus lift procedures in the atrophic posterior maxilla with deficient bone can be demonstrated by the proper selection of cases [6].

During the sinus lift procedures, different graft materials are used to increase the height of the bone in the sinus. Although gold-standard bone grafts are autogenous bone grafts, they have certain limitations, including high morbidity, more surgical sites, consent of the subjects, and an additional second surgical site [7]. Platelet-rich fibrin (PRF) is a bio-graft material that is an autologous fibrin matrix with leukocyte and platelet growth factors that stimulate the differentiation and proliferation of osteoblasts, showing that PRF has regenerative potential. PRF use while sinus elevation can enable prompt healing before implants are functionally loaded. Additionally, PRF acts as a filler material to prevent the sinus membrane seal recoil [8].

PRF has various advantages, including minimal donor site morbidity, cost-effectiveness, increased angiogenesis by multiplication of osteoblasts and fibroblasts, and local supply of growth factors [9]. Hence, this study was conducted to evaluate the outcomes of indirect sinus lift with hydraulic pressure and simultaneous placement of an implant using PRF.

## Materials and methods

This study aimed to evaluate the outcomes of indirect sinus lift with hydraulic pressure and simultaneous placement of an implant using PRF. The study was carried out after obtaining clearance from the concerned ethical committee (Department of Oral and Maxillofacial Surgery, Baba Jaswant Singh College of Dental Sciences Hospital and Research Institute issued approval ICMCH/2020/19). The study subjects were patients coming to the Department of Oral and Maxillofacial Surgery of the Institute for the placement of dental implants to replace missing teeth.

The study included 24 subjects, both males and females, in the age range of 18-74. Subjects with no medical history; those with one or more missing maxillary premolars, first molar, or second molar with the alveolar ridge of 8 mm height on cone-beam computed tomography (CBCT) or orthopantomogram (OPG); those with low sinus; and those willing to consider dental implants as a tooth replacement option were included in the study. The exclusion criteria were subjects with contraindications to surgery/local anesthesia, those with chronic nasal obstruction/sinusitis, immunocompromised subjects, uncontrolled diabetics, and those with a bone height of 4 mm.

**Figure 1:**
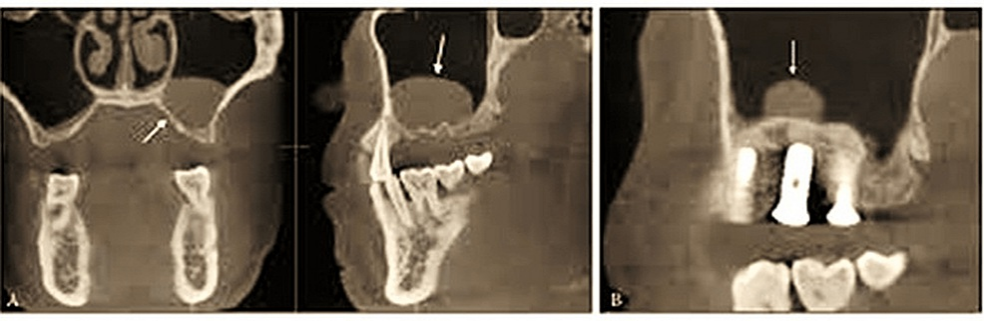
CBCT image of the region. A: sinus lift after the hydraulic system; B: bone fill. CBCT: cone-beam computed tomography

After final inclusion, OPG and CBCT (Figure 1) were performed to assess residual bone height at the implant placement site from the sinus floor to the alveolar crest along with routine blood investigations. There is a proper bone gain after the lift and bone loss.

**Figure 2:**
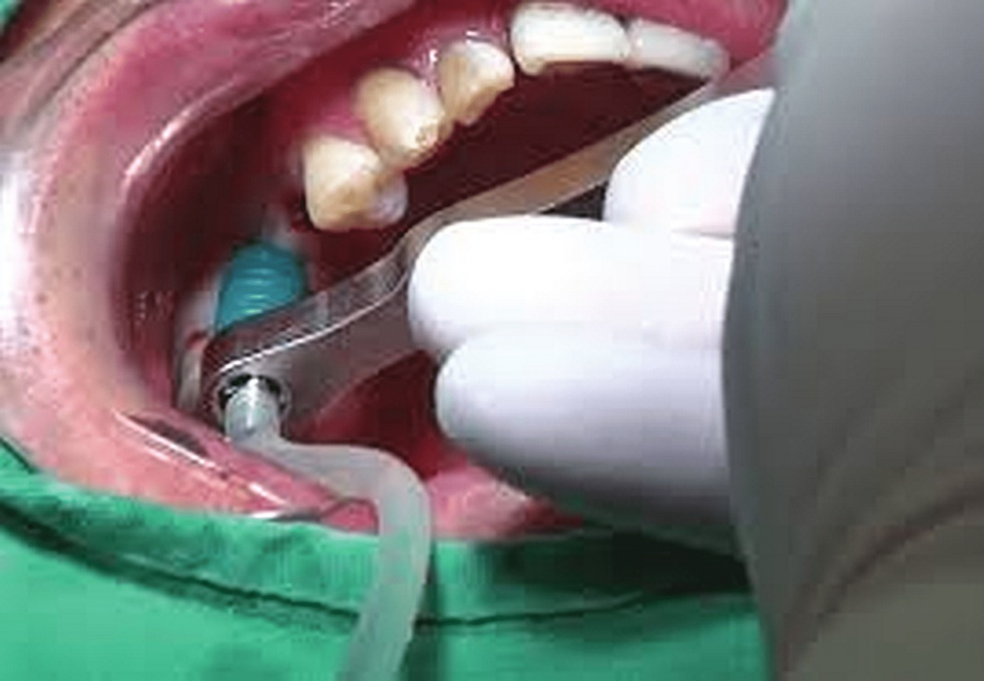
Hydraulic system for the sinus lift.

The nasal decongestant was started a day before surgery. To prepare PRF, 10 mL of intravenous blood was withdrawn under aseptic and sterile conditions in a test tube without anticoagulants and was centrifuged at 2,700 rpm for 12 minutes following Choukroun’s procedure. The resultant PRF was used as space-filling material as well as the membrane that was transferred onto Schneider’s membrane. The hydraulic lift was placed, as shown in Figure 2.

After preparing the implant site, a saline ejector was placed. To prevent membrane tear, 5 mL syringe saline was pushed by lifting the membrane using the hydraulic pressure followed by implant placement. PRF clots of gelatinous consistency that were cut into small pieces under an elevated membrane were placed to give a cushion effect while placing dental implants, and the site was closed with sutures.

Postoperatively, antibiotics and analgesics along with 0.2% chlorhexidine mouthwash were given to all the subjects for five days, and the postoperative evaluation was done concerning bone density and bone height at six months to formulate the results.

## Results

The mean age of the subjects in the present study was 39.82 ± 8.44 years. The maximum number of subjects were in the age group of 41-50 years (50%, n = 12), followed by 31-40 years (29.16%, n = 7), 18-30 years (16.6%, n = 4), and the least in the >50-year age group (4.16%, n = 1). There were 66.6% (n = 16) males and 33.3% (n = 8) females in this study (Table 1).

**Table 1:** Demographic characteristics of the study subjects.

Characteristics	Percentage (%)	Number (n)
Age range (years)		
18–30	16.6	4
31–40	29.16	7
41–50	50	12
>50	4.16	1
Mean age (years)	39.82 ± 8.44	
Gender		
Males	66.6	16
Females	33.3	8

The study included 24 subjects with eight females and 16 males, and the included teeth were 10 right first maxillary molars and eight left maxillary first molars, two right maxillary second premolars, two left maxillary first premolars, and two left maxillary second premolars. For all teeth, residual ridge height was measured preoperatively as well as postoperatively. The average mean height preoperatively was 5.573 ± 0.66 mm which showed a significant increase postoperatively to 9.603 ± 0.78 mm. The mean difference was found to be statistically significant (p < 0.001), as shown in Table 2.

**Table 2:** Preoperative and postoperative residual ridge height in the study subjects. M: male; F: female

Serial number	Gender	Preoperative residual ridge height (mm)	Postoperative residual ridge height (mm)	P-value
1	M	5.9	10.3	-
2	M	6.4	9.7	-
3	F	5.3	9.4	-
4	M	5.5	9.5	-
5	M	4.7	10.3	-
6	F	4.9	10.1	-
7	F	5.3	10.4	-
8	M	5.2	7.7	-
9	M	7.2	10.7	-
10	M	6.6	8.4	-
11	F	5.2	8.1	-
12	M	5.4	9.7	-
13	M	5.7	10.2	-
14	M	5.4	10.4	-
15	F	4.6	9.6	-
16	F	5.2	10.2	-
17	M	5.9	10.3	-
18	M	6.5	9.4	-
19	M	6.4	10.3	-
20	M	6.2	9.4	-
21	F	5.2	7.7	-
22	M	6.6	8.4	-
23	F	6.4	9.7	-
24	M	4.6	9.6	-
25	Mean value	5.573 ± 0.660041	9.603 ± 0.781679	<0.001

On assessing the maximum sinus membrane lift postoperatively, the mean sinus membrane lift seen in 24 study subjects was 4.8 ± 2.2 mm, as presented in Table 3.

**Table 3:** Evaluation of maximum sinus membrane lift in the study subjects postoperatively. M: male; F: female

Gender	Preoperative residual ridge height (mm)	Implant size length (mm)	Effective sinus membrane lift
M	5.9	10	4.3
M	6.4	11.5	5.2
F	5.3	10	4.8
M	5.5	10	4.8
M	4.7	10	5.2
F	4.9	10	5.1
F	5.3	10	4.8
M	5.2	10	5.1
M	7.2	11.5	4.4
M	6.6	11.5	4.8
F	5.2	10	4.5
M	5.4	10	4.9
M	5.7	10	4.5
M	5.4	10	4.7
F	4.6	10	5.1
F	5.2	10	5.1
M	5.9	10	4.3
M	6.5	11.5	5.1
M	6.4	11.5	5.2
M	6.2	10	4.1
F	5.2	10	5.2
M	6.6	10	5.2
F	6.4	10	4.8
M	4.6	10	4.8

Assessment of implant stability immediately postoperatively and after six months was assessed using the Osstell device, and it was seen that for all 24 subjects, the Implant Stability Quotient (ISQ) was significantly higher at six months postoperatively compared to immediately after the procedure. The mean ISQ value was 69.07 ± 3.39 when assessed immediately postoperatively using the Osstell device, whereas the ISQ was 72.92 ± 2.714 when assessed at six months after the procedure. This difference was statistically non-significant (p < 0.001), as shown in Table 4.

**Table 4:** Assessment of implant stability immediately postoperatively and after six months. M: male; F: female

Gender	Implant Stability Quotient (immediately postoperatively)	Implant Stability Quotient (six months postoperatively)	P-value
M	72	72	-
M	70	75	-
F	70	75	-
M	70	72	-
M	67	71	-
F	66	70	-
F	67	69	-
M	65	68	-
M	65	75	-
M	66	76	-
F	70	73	-
M	73	74	-
M	70	72	-
M	71	70	-
F	72	70	-
F	74	75	-
M	73	72	-
M	61	75	-
M	67	78	-
M	72	76	-
F	66	70	-
M	70	73	-
F	61	75	-
M	65	68	-
Mean value	69.07 ± 3.396	72.92 ± 2.714	<0.001

## Discussion

The consensus reports on sinus augmentation with simultaneous grafting suggest that to achieve the orthoalveolar form, segmental alveolar bone reconstruction is a favorable treatment modality to maintain bone and gingival health. Alveolar bone reconstruction with simultaneous grafting is an accepted technique to restore the lost architecture of the crestal bone, establish the bolus deflection, improve the emergence profile esthetics, and increase the bone for sinus augmentation [10].

The mean age of the study subjects was 39.82 ± 8.44 years. In the age group of 41-50 years, there were 50% (n = 12) subjects, followed by 29.16% (n = 7) of the subjects in the 31-40-year age group, 16.6% (n = 4) of the subjects in the 18-30-year age group, and 4.16% (n = 1) of the subjects in the >50-year age group. There were 66.6% (n = 16) males and 33.3% (n = 8) females in the study. The data of the study subjects were similar to the studies of Yilmaz et al. [10] and Tajima et al. [11], which evaluated subjects with demographics similar to the present study. Nearly half of the study subjects were in the age range of 41-50 years, showing that this is the most common age for the loss of maxillary posterior teeth. Higher tooth loss was seen in male subjects compared to females, which was also consistent with other studies by Tajima et al. [10] and Yilmaz et al. [11].

The PRF is better than other bone graft materials as it contains high levels of growth factors which plays a central role in the bone healing process and hemostasis with an established role in bone defect healing and bone regeneration. The average mean height preoperatively was 5.573 ± 0.66 mm which showed a significant increase postoperatively to 9.603 ± 0.78 mm. The mean difference was found to be statistically significant (p < 0.001). These findings were consistent with those of Kanayama et al. [12] and Moussa et al. [13], which reported a significant increase in residual alveolar ridge height following indirect sinus lift and simultaneous placement of implant using PRF. This shows the efficacy of simultaneous implant placement, bone grafts, and PRF use in the cases with the atrophic posterior maxilla with the increase in the vertical height of the alveolar bone following augmentation and PRF use. The consensus comparing different bone graft materials with PRF or without PRF states that PRF alone has equivalent results compared to PRF along with bone graft materials [12].

Indirect sinus lift using hydraulic pressure technique has advantages over other methods of sinus lifting including predictable implant survival rates, lesser resorption, better placement of bone grafts, acceptable bone healing, simultaneous implant placement possibility, lesser membrane rupture, and is a more conservative approach. On assessing the maximum sinus membrane lift in the study, the mean sinus membrane lift seen in 24 study subjects was 4.8 ± 2.2 mm. These results are in agreement with the studies of Tanaka et al. [14] and Xiu et al. [15], which reported comparable maxillary sinus membrane lift after indirect sinus lift and simultaneous placement of implants using PRF, as seen in the present study. The sinus membrane lift was seen in the present study. It showed that the use of bone grafts along with the PRF membrane is an efficacious treatment modality in cases with an atrophic posterior maxilla.

The ISQ was significantly higher at six months postoperatively compared to the ISQ immediately after the procedure. The mean ISQ value was 69.07±3.39 when assessed immediately postoperatively using the Osstell device, whereas, the ISQ was 72.92 ± 2.71 when assessed six months after the procedure. This difference was statistically non-significant (p < 0.001). These results are consistent with those of Nedir et al. [16] and Khairnar et al. [17], which reported increased stability following the indirect sinus lift and bone augmentation postoperatively. The ISQ showed an increase six months postoperatively, depicting that stability of the dental implants placed was higher following the sinus membrane augmentation using bone graft and PRF, leading to better stability of the dental implants in the posterior atrophic maxilla compared to dental implants placed without augmentation of the sinus membrane.

The study has a few limitations and improvement scope, including a larger number of patients, sample size, and longer assessment in the prospective study setting.

## Conclusions

Considering its limitations, from its findings, the present study concludes that minimally invasive indirect sinus lift with bone augmentation using PRF leads to a significant postoperative increase in residual alveolar ridge height and implant stability with fewer postoperative complications compared to other techniques of sinus lift in the posterior maxillary region.
